# 

**Published:** 1852-03

**Authors:** 


### ARTICLE VIII.

### NOTICE TO SUBSCRIBERS—EXTENSION OF TIME.

As many of our subscribers have written to ascertain the amout of their dues, and it is impossible for them to pay up before the close of the time specified in our last number, during which we would take two dollars per volume in full liquidation of our claims, we have concluded to send with this number, the bills of subscribers made out according to this proposition, and extend the time to the 15th of May next, when if not paid, the additional one dollar per volume for volumes 2, 3, & 4 of the N. W. Med. & Surg. Jour. will be added to the accounts, and the whole placed in the hands of an Attorney for collection.

Please remit, Post-paid, at our risk, and receipts shall be acknowledged, as heretofore.

### INDEX.

Aconitum Napellus, by J. A: Preston, M. D. 415
Address before the Ills. State Medical Society, by Prof. Herrick, 202
Alabama Med. Assoc'n, notice of 57
Allaire on Chloroform in Neuralgia 388
Ames on extraction of child by a novel process 193
Amer. Med. Assoc'n Minutes of 115
" " " Notice of transactions of 157, 124, 421
Amer. Med. Assc'n Meeting of 445
Analysis of the Tomato by Dr. Plummer 78
Aneurism of Aorta similating Phthisis 343
Aurora Borealis, singular influence of by R. S. Miller Esq. 253
Banks' case of supposed violence or Poisoning 46
Beale on laws of health, notice of 282, 325
Beck on infant Therapeutics, notice of 340
Bernard on the office of the Lacteals 137
Biddle on poisoning from Opium 146
Bird on Urinary deposits, etc. notice of 330
Bracket on Inocculation of Morphine in Sciatica 190
Brainard on Enlargement of Ethmoidal Cell 408
Brainard on Ununit'd fracture of Femur 410
" " Ligature of Femoral artery 414
" " Dislocation of Femur in Thyroid Fossa 418
Bullard on Puerperal Fever at Indianapolis, Ia. 99
Carpenters' Physiology, notice of 324
Cass co., Mich. Medical Society 219
Castleman on Chloroform in Erysipelas 192
Catrptric Test for Cataract 73
Central Med. Society of Illinois 104
Cerebro Arachnitis by Dr. Jas. Smick 235
" " by J. B. Nash M.D. 319
" Spinal Arachnitis, by J. S. Whitmire M. D. 382
Churchill's Midwifery, notice of 280
Chiniodine in Intermittent Fever 65
Chloroform in Erysipelas 59
" " Scarlatina 76
Cholera, 161
Chloride of Sodium as used by Prof. Herrick, 193
Cod Liver Oil in nursing sore mouth by by Prof Evans 190
Collodion in Erysipelas etc., translated from the French by J. P. Lynn 307
Clark on Chloroform in Insanity 103
Clinical Lectures by Prof. Davis, 1, 162, 265 292
Collodion in Small Pox etc. by E M'Arthur M. D. 258
Compression of the Aorta for Uterine Hemorrhage by I. E. Oatman M. D. 33
Cragie's General Pathology, notice of 322
Davis' Clinical Lectures in Hospital, 1, 162 265, 392
Davis' Experiments on Assimilation 169
" History & Reviewing in gen'l 17
" Introductory Lecture, 298
" " " notice of 459
Drake's Discourses, notice of 471
Death of distinguished Physicians 68
Diet in protracted Fevers 360
Dislocation and Fractures of the joints, by Cooper, notice of 113
Dislocation of Femur, new mode of reduction by Prof. Brainard. 418
Dislocation of Femur, new mode of reduction, by Dr. Reid, 445
Dissector, Wilson's, notice of 112
Dowler, Prof. B., and the British and Foreign Review 457
Dysentery, Morphine in, by Dr. Tullis 149
Electic Jour. of Education, notice of 463
Esysipilas, Chloroform in; by A.S. Casman, M. D. 192
Enlargement of an Ethmoidal Cell by Prof. Brainard 408
Eruptions of the skin during Pregnancy 70
Evans on use of Obstetrical Extractor 40
" " Pyroligneous acid as a gargle 419
" " Cod Liver Oil in Nursing Sore mouth, 199
Experimental Researches by B. Dowler M. D, notice of 220
Experiments on an Alliga.or, 446, 452
Free Medical Schools, 378
Fulton co. Ills. Med. Society 109
Goff on Poisoning with Mercury 406
Gregory on Animal Mag'tism, notice of 283
Gross on Urinary Organs, notice of 281
Hathway on Inunction in Rubeola etc. 262
Hermaphrodism, a case of 293
Hendricks on Puerperal convulsions, 35
Herrick's Address before State Med. Society. 202
Herrick on the mortality of Chicago 38
Herrick on use of chloride of Sodium 193
Hewson on diameters of Fœtal head 290
Hints on the Med. Profession, notice of 332
History of Med. Education, Reviewing in General, by Prof. Davis. 17
Homœopathy and Royal College Phys. 460
Hooker on Homœopathy, notice of 429
Hot water in spasms 59
Hull's treatment of Puerperal convulsions 48
Illinois State Med Soc'ty 81, 152, 272
Indiana Hospital for the Insane 371, 467
Insanity cured by Chloroform 103
Introductory Address, notice of 159
Introductory Lecture by Prof Davis 298
" " notice of 459
Johnson's translation of Von Mauthener on extract of Beef's Blood 260
Keineispathy, 363
Lattimore on Digestion 285
Ligature of Femoral Artery, by Prof. Brainard 414

### INDEX.

Lynn's Translation of Collodion in Erysipelas 307
Meclaise's Surgical Anatomy, notice of 323
Malgaigne's Surgery, notice of 332
Matthews on Tobacco in Inflammation 315
McArthur on Collodion in Small Pox 258
McGirr on Rattle Snake bite 311
Medical Delusions by Hooker, notice of 50
Microscopist, notice of 331
Miller on Aurora Borealis 253
Miscellaneous Intelligence, 231,304,379 472
Morelands Extracts from Boston Society for Medical Improvement 295
Morris on Scarlet Fever, notice of 432
Morton S. G. notice of death of 141
Mountain and Malarious Fevers by Ira E. Oatman M. D. 105
Murphys Chemistry, notice of 46
Maygatt on Occlusion of Os Uteri 255
Nash on Cerebro-Arachnitis 319
Notice to Subscribers, 471
Neil's case of Hermaphrodism 293
New Arrangement 381
New Postage Law 161
New Theory of Respiration 446
Neuralgia cured by Chloroform, by P. A. Allaire, M. D. 388
Notice to Readers, etc. 307
Oatman on compression of the Aorata 33
" " Mount'n & Malar'us fevers 105
Obituary Notices, 161,233,306,380
Observations on the use of Obstetrical Extractor, by Prof. Evans, 40
Occlusion of Os Uteri by E. G. Maggatt, M. D. 256
Ovarian Irritation, Churchill on 350
Ovary Removed for a Labial Cyst 297
Paralysis of Muscles of Deglutition 341
Physicians Prescription Book, notice of 111
Poisoning by Arsnic, by S. Thompson M. D. 89
Poisoning with Per Chloride of Mercury, by W. W. Goff M. D. 406
Poisoning, Case of supposed, by E. C. Banks M. D. 46
Pocket formula, notice of 336
Physicians Visiting List, notice of 339
Practical Items 366
Preston on Aconitum Napellus 415
Puerperal Convulsions, by J. A. Preston M. D. 216
Puerperal Convulsions, by J. E. Hendrick's M. D. 35
Puerperal Fever, by T. Bullard, M. D. 99
Pure Drugs and Medicines, 229
Pyro-Ligneous Acid as a Gargle, by Prof. Evans, 419
Quackery in America, 345
Rankings Abstract, notice of 324
Rattle Snake Bite, a case of, by J. E. McGirr, M. D. 311
Rubeola, a case of, by O. P. Hathway, M. D. 262
Rush Medical College 380
" " " Commencement of 469
Sciatica treated by Inoculation of Morphine, by C. Brackett, M. D. 190
Smick on Cerebro Arachnitis 234
Statistics of Medical Schools 84
Talcott's Address, notice of 464
Tilt on Menstruation, notice 114
Tobacco in Inflammation, by W. Mathews, M. D. 315
The Journal 303
The Medical Profession & Public Press 465
Thompson on Poisoning by Arsnic, 89
Transactions of the Med. Associa'n of Southern Central N. Y., notice of 466
Transfusion of Blood 444
United States Pharmacopœia, notice of 325
Ununited Fracture treated by Sub-cutaneous Perforation of the Bone, by Prof. Brainard 410
Vaccination, Protective power of 356
Valedictory, 461
Walker on Intermarriage, notice of 337
Welfare of the Medical Profession, 459
Western Medico Chirurgical Journal 228
Whiteside co. Medical Society, 264
Whitmore on Cerebro.Arachnitis 382

### NOTICE TO READERS AND CORRESPONDENTS.

An original paper has been received from Dr. Hatheway, which we hand over to our successors.

Many Addresses, Catalogues and Papers have been received, which our space will not allow us to notice.

The following Journals are received in exchange:

The American Journal of Medical Sciences.
British and Foreign Medico-Chirurgical Review.
Ranking's Half Yearly Abstract.
The American Journal of Pharmacy.
The Medical Examiner, Philadelphia.
The New Orleans Medical and Surgical Journal.
The American Journal of Insanity, Utica, N. Y.
The New York Journal of Medicine and Collateral Sciences.
Buffalo Medical Journal.
Charleston Medical Journal and Review.
The American Journal of Dental Sciences.
Western Journal of Medicine and Surgery.
Western Lancet and Hospital Reporter.
Western Medico-Chirurgical Journal.
St. Louis Medical and Surgical Journal.
The Stethoscope.
Northern Lancet.
Ohio Medical and Surgical Journal.
Transylvania Medical Journal.
Boston Medical and Surgical Journal.
Medical News and Library, Phild.
Newhampshire Journal of Medicine.
New Jersey Medical Reporter.
New Orleans Monthly Medical Register.
London Lancet.
Southern Medical and Surgical Journal, Ga.
The New York Monthly Law Magazine.

Several Quack Medical Journals and many newspapers, have been received, which we have not room to name, nor desire to exchange with.

### RECEIPTS

FOR THE JOURNAL FOR THE MONTHS OF JANUARY AND FEBRUARY, 1852.

### ILLINOIS.

| | | |
|---|---|---|
| Drs. | J. M. Mayfield, | $2,00 |
| | J. W. McKinney, | 1,00 |
| | T. S. Mills, | 4,00 |
| | H. Noble, | 2,00 |
| | R. Trowbridge, | 2,00 |
| | P. Dickson, | 4,00 |
| | J. H. Gibson, | 4,00 |
| | F. Blades, | 2,00 |
| | R. Robinson, | 2,00 |
| | J. Woodworth, | 2,00 |
| | B. K. Hart, | 2,00 |
| | J. Harwood, | 10,00 |
| | J. J. Lescher, | 2,00 |
| | C. J. Miller, | 2.00 |
| | G. Ryon, | 2,00 |
| | C. A. Hathwell, | 2,00 |
| | F. P. Wright, | 4,00 |
| | T. Hoffman, | 2,00 |
| | J. C. Hinsey, | 2,00 |
| | S. H. Gaston, | 10,00 |
| | Thos. Hale, | 5,00 |
| | W. T. Briscoe, | 6,00 |
| | Pomeroy & Stough, | 2,00 |
| | S. C. Hatch, | 5,00 |
| | D. Ransom, | 2,00 |
| | H. W. Hopkins, | 2,00 |
| | G. A. Bunker, | 2,00 |
| | F. B. Ives, | 2.00 |
| | Duff Green. | 8,00 |
| | S. H. Vriedenburgh, | 3,00 |
| | F. T. Miner, | 5,00 |
| | J. H. Brown, | 2,00 |
| | J. N. Abell, | 2,00 |
| | L. Nutting, | 2,00 |
| | L. A. Winslow, | 2,00 |
| | S. O. Long, | 2,00 |
| | D. Ray, | 2,00 |
| | J. J. Fisher, | 2,00 |
| | J. K. Leedy, | 2,00 |
| | Ira B. Curtis, | 2,00 |
| | Thos. A. Brown, | 2,00 |
| | C. J. Corbin, | 5,00 |
| | J. B. Nash, | 2,00 |

### INDIANA.

| | | |
|---|---|---|
| Drs. | W. G. Montgomery, | $6,00 |
| | A. L. Pettijohn, | 3,00 |
| | T. Newkirk, | 4,00 |
| DRS. | T. B. Thompson, | $5,00 |
| | W. H. Gifford, | 2,00 |
| | L. Dunlap, | 6,00 |
| | J. P. Russell, | 4,00 |
| | M. W. Gentry, | 4,00 |
| | B. R. Westfall, | 2,00 |
| | G. M. Huggans, | 5,00 |
| | J. A. Martin, | 2,00 |
| | A. M. Porter, | 2,00 |
| | R. Faber, | 8,00 |
| | J. Pennington, | 4,00 |
| | R. C. Bond, | 4,00 |
| | G. A. Durr, | 6,00 |
| | H. W. Mann, | 4,00 |
| | S. K. Hardy, | 2.00 |
| | S. B. Harriman, | 8,00 |
| | R. D. Manzy, | 2,00 |
| | Roberts & Hoyne, | 2,00 |
| | C. B. Richards, | 4,00 |
| | W. Bamford, | 4,00 |
| | C. West, | 2,00 |
| | W. A. Barns, | 2,00 |
| | H. Dyer, | 4,00 |

### WISCONSIN

| | | |
|---|---|---|
| DRS. | J. Ingersoll, | 2,00 |
| | M. B. Goff, | 2,00 |
| | A. Sampson, | 3,00 |
| | John S. Schofield, | 2,00 |
| | C. Miller, | 2,00 |
| | D. L. Downs, | 2,00 |
| | B. G. Collins, | 2,00 |
| | C. F. Pease, | 2,00 |
| | D. M. Cruthers, | 2,00 |
| | O. D. Coleman, | 2,00 |

### MICHIGAN.

| | | |
|---|---|---|
| DRS. | B. P. Wells, | 3,00 |
| | A. R. Calkins, | 4,00 |
| | F. Myers, | 4,00 |
| | R. Stephenson, | 2.00 |

### IOWA.

| | | |
|---|---|---|
| DRS. | H. L. Whitman, | 6,00 |
| | F. W. Coolidge, | 2,00 |

### OHIO.

| | | |
|---|---|---|
| DRS. | W. W. Jones, | 2,00 |
| | W. H. Baldwin, | 4,00 |

### NEW YORK.

| | | |
|---|---|---|
| DRS. | R. Scott, | 2,00 |
| | T. S. Loomis, | 1,00 |

### PROSPECTUS OF VOL. V.

OF

### THE NORTH WESTERN MEDICAL AND SURGICAL JOURNAL

EDITED BY

**W. B. HERRICK, M. D.,**
Prof. of Anatomy, etc. in Rush Medical College; Surg. to the U. S. Marine Hospital and one of the Surgeons to the Illinois General Hospital, etc.

ASSISTED BY

**H. A. JOHNSON, M. D.,**

---

*The North Western Medical and Surgical Jorrnal*, will be published hereafter, on the first of each month, beginning in May, in numbers each containing exclusive of advertisements, 48 pages closely printed, with new type ordered expressly for this work; thus presenting at the end of the year a volume of nearly Six Hundred pages.

The change from a bi-monthly to a monthly Journal has been made in accordance with the expressed wishes of numerous subscribers.

The Editor, with his associate, Dr. JOHNSON, who will devote his time exclusively to the Journal, will endeavor in addition to a full and complete Domestic Summary, to present numerous extracts from English and translations from French and German Medical periodicals.

In addition to the Communications from Contributors, whose favors it is hoped will be contiuued, the Original Department will contain from time to time, reports of Cases treated, and of Observations made, in the two Hospitals, now fully organized in Chicago

---

### TERMS.

### Two Dollars Per Annum in Advance.

---

As an inducement to Physicians and others to act as Agents, in extending the circulation of the Journal, Three copies will be furnished to new subscribers for $5,00

| | | | | | |
|---|---|---|---|---|---|
| Five | " | " | " | " | $8,00 |
| Seven | " | " | " | " | $10,00 |

---

*TERMS OF ADVERTISING.*

| | | | |
|---|---|---|---|
| 1 Page, 1 year, | $20,00 | 1 Page single insertion, | $5,00 |
| ½ " " | $12,00 | ½ " " " | $3,00 |

☞ Letters must be Post-paid, and directed to the "North-Western Medical and and Surgical Journal, Chicago, Ills." A list of receipts will appear in each number.

*Chicago, March* 1*st*, 1852.

